# Potential surrogate endpoints for overall survival in locoregionally advanced nasopharyngeal carcinoma: an analysis of a phase III randomized trial

**DOI:** 10.1038/srep12502

**Published:** 2015-07-29

**Authors:** Yu-Pei Chen, Yong Chen, Wen-Na Zhang, Shao-Bo Liang, Jing-Feng Zong, Lei Chen, Yan-Ping Mao, Ling-Long Tang, Wen-Fei Li, Xu Liu, Ying Guo, Ai-Hua Lin, Meng-Zhong Liu, Ying Sun, Jun Ma

**Affiliations:** 1 Sun Yat-sen University Cancer Center, State Key Laboratory of Oncology in South China, Collaborative Innovation Center of Cancer Medicine, Guangzhou, People’s Republic of China; 2 Department of Radiation Oncology, Cancer Center, The First People’s Hospital of Foshan, Foshan, People’s Republic of China; 3 Department of Radiation Oncology, Fujian Provincial Tumor Hospital, Fuzhou, People’s Republic of China; 4 Department of Medical Statistics and Epidemiology, School of Public Health, Sun Yat-sen University, Guangzhou, People’s Republic of China

## Abstract

The gold standard endpoint in trials of locoregionally advanced nasopharyngeal carcinoma (NPC) is overall survival (OS). Using data from a phase III randomized trial, we evaluated whether progression-free survival (PFS), failure-free survival (FFS), distant failure-free survival (D-FFS) or locoregional failure-free survival (LR-FFS) could be reliable surrogate endpoints for OS. Between July 2002 and September 2005, 316 eligible patients with stage III-IVB NPC were randomly assigned to receive either radiotherapy alone or chemoradiotherapy. 2- and 3-year PFS, FFS, D-FFS, and LR-FFS were tested as surrogate endpoints for 5-year OS using Prentice’s four criteria. The Spearman’s rank correlation coefficient was calculated to assess the strength of the associations. After a median follow-up time of 5.8 years, 2- and 3-year D-FFS and LR-FFS were not significantly different between treatment arms, in rejection of Prentice’s second criterion. Being consistent with all Prentice’s criteria, 2- and 3-year PFS and FFS were valid surrogate endpoints for 5-year OS; the rank correlation coefficient was highest (0.84) between 3-year PFS and 5-year OS. In conclusion, PFS and FFS at 2 and 3 years may be candidate surrogate endpoints for OS at 5 years; 3-year PFS may be more appropriate for early assessment of long-term survival.

Nasopharyngeal carcinoma (NPC) is a unique head and neck cancer that is relatively common in southern China, with a yearly incidence of 15 to 50 cases per 100,000^1^. Radiotherapy (RT) is the primary treatment for non-metastatic NPC: control of early-stage disease with RT alone is usually favorable; however, the response of locoregionally advanced NPC to RT alone is unsatisfactory^2^. The efficacy of combined chemotherapy and RT have been investigated in numerous trials^3^. We performed a phase III randomized clinical trial of concurrent chemoradiotherapy with adjuvant chemotherapy (CRT) vs. radiotherapy alone in locoregionally advanced NPC between July 2002 and September 2005. The preliminary results were published in 2008^4^, and 5-year follow-up in 2013^5^. Both analyses found the experimental arm lead to significantly better overall survival (OS) than the control arm. The CRT therapeutic regimen established in this trial is now considered a standard of care for locoregionally advanced NPC^6^.

OS is the gold standard endpoint in trials for NPC; 5-year OS is commonly used to assess the long-term benefits of a treatment. OS is easily and reliably measured and is easy to interpret. However, analysis of OS requires a large sample size and long follow-up duration to detect significant differences; changes in OS may also be diluted by non-cancer deaths and administration of subsequent treatments after progression.

Identification of surrogate endpoints for OS would shorten trial duration and thus speed up the identification of effective treatments for NPC. Ideally, such surrogates should be based on clinical information that is available as soon as possible after definitive therapy. In NPC trials, progression-free survival (PFS) and failure-free survival (FFS) represent potential surrogate endpoints; distant failure-free survival (D-FFS) and locoregional failure-free survival (LR-FFS) may also serve as valid surrogates. To the best of our knowledge, no study has yet validated these endpoints at an early time-point (e.g., 2 and 3 years) as surrogates for long-term survival (e.g., 5-year OS) in locoregionally advanced NPC. To establish an endpoint as a surrogate, a set of statistical conditions, known as Prentice’s criteria, need to be met^7^. The purpose of this analysis was to evaluate whether 2- and 3-year PFS, FFS, D-FFS or LR-FFS (as defined below) have potential as surrogate endpoints for 5-year OS according to Prentice’s criteria based on data from our phase III randomized trial.

## Methods

### Patients, treatment and follow-up

The data used in this analysis was based on the trial progress report^5^ and was extracted from the trial database on February 25, 2011. The study was approved by the Institutional Review Board of Sun Yat-sen University Cancer Center, and was conducted in accordance with the Good Clinical Practice guidelines; all patients provided written informed consent before treatment.

The study cohort consisted of 316 eligible patients (158 patients randomly assigned to each group). Patients were eligible for the trial if they met the following inclusion criteria: (1) histologically-proven, non-keratinizing or undifferentiated NPC (World Health Organization type II or III); (2) stage III-IVB (T3-4NxM0 or TxN2-3M0) disease according to the 1997 American Joint Commission on Cancer staging system; (3) an Eastern Cooperative Oncology Group system performance status of 0–2; (4) adequate renal function, as demonstrated by a creatinine clearance rate of at least 60 mL/min; and (5) adequate hematologic function, as demonstrated by a leukocyte count ≥4,000/ μL and platelet count ≥ 100,000/ μL. Patients were excluded for any of the following exclusion criteria: keratinizing squamous cell carcinoma or adenocarcinoma; age ≥70 or ≤16 years; pregnancy or lactation; history of renal disease; unstable cardiac disease requiring treatment; a history of previous radiotherapy or chemotherapy; or a history of prior malignancy.

All enrolled patients received 2-dimensional RT (2 Gy fractions 5 five times per week). The cumulative doses were >66 Gy to the gross tumor, 60 to 66 Gy to the involved areas of the neck and >50 Gy to the uninvolved areas. For the CRT group, concurrent chemotherapy with weekly cisplatin (40 mg/m^2^ on day 1) was given intravenously for 7 weeks during the RT phase. Three subsequent adjuvant chemotherapy cycles, consisting of cisplatin (80 mg/m^2^ intravenously) on day 1 and fluorouracil (120 h intravenous infusion; 800 mg/m^2^ daily) on days 1–5 were administered during weeks 5, 9 and 13 after the completion of RT. There were no dose modifications during concurrent chemotherapy. Adjuvant chemotherapy dose modifications were based on nadir blood counts and the interim toxicities of the preceding cycle. Further details are provided in the trial progress report^5^. Whenever possible, patients with documented relapse or persistent disease received salvage treatments (re-irradiation, chemotherapy, surgery) according to institutional guidelines.

Patients attended follow-up visits every 2 months in year 1, every 3 months in the subsequent 2 years, and then every 6 months thereafter (or until death). All local recurrences were diagnosed by fiberoptic endoscopy and biopsy or MRI/intensive CT of the nasopharynx and the skull base. Regional recurrences were diagnosed by clinical examination of the neck; irresolute cases were confirmed by fine needle aspiration or MRI/intensive CT scans. Distant metastases were diagnosed by clinical symptoms, physical examinations and imaging methods including chest X-ray, bone scan, MRI, CT and abdominal sonography.

### Study endpoints

The major trial endpoint was 5-year OS (time from randomization to death from any cause). The primary potential surrogate endpoints in this study were 2- and 3-year PFS and FFS; PFS was defined as the time from randomization to failure or death from any cause, FFS as the time from randomization to the first failure at any site. Additionally, we also examined secondary potential surrogate endpoints: 2- and 3-year D-FFS and LR-FFS, which were defined as time from randomization to locoregional or remote failure, respectively. Patients with no documented evidence of events were censored at the date of last follow-up; all events that occurred after 2 or 3 years were also censored.

### Statistical analysis

Prentice’s four criteria^7^ were used to assess the validity of the surrogates. A surrogate for a true endpoint should yield a valid test of the null hypothesis that no correlation exists between the treatment and the true response. The following four criteria were tested to validate Prentice’s criteria: 1) the treatment is significantly prognostic for the true endpoint (i.e., 5-year OS); 2) the treatment is significantly prognostic for the surrogate endpoint (e.g., 2- and 3-year PFS, FFS, D-FFS or LR-FFS); 3) the surrogate endpoint is significantly prognostic for the true endpoint; and 4) the full effect of the treatment on the true endpoint should be captured by the surrogate endpoint. The incidence rates for the true and surrogate endpoints were estimated using the Kaplan-Meier method and compared using the log-rank test^8^. Adjusted Cox proportional hazard models were used to determine hazard ratios (HRs) for the two arms with respect to each survival outcome^9^. The following parameters were included as covariates for each analysis: age (>50 vs.≤50 years), sex (male vs. female), T classification (T3–4 vs. T1–2), N classification (N1–3 vs. N0) and treatment arm (CCRT + AC vs. RT) for Prentice’s criteria 1, 2 and 4, and surrogate endpoints (with vs. without events ≤2/3 years) for Prentice’s criterion 3. Additionally, the Spearman rank correlation coefficient (ρ) for the distributions of the candidate surrogate endpoints and 5-year OS at the individual level were calculated using a bivariate survival model^10^ to assess the strength of the associations as ρ^2^ reflects the amount of variation explained by the surrogate. SPSS 19.0 software (IBM, Armonk, NY, USA) was used for all analysis.

## Results

Between July 2002 and September 2005, 316 eligible patients were randomly assigned to the treatment arms in the phase III trial (158 patients in each group). Median follow-up for the entire cohort was 5.8 years (range, 0.1–8.5 years). Pretreatment characteristics were well-balanced between the two arms (Table 1). It should be noted that there were 61 patients with a T1-2 classification in our trial, of whom eight (13.1%) had a T1 classification and 53 (86.9%) had a T2 classification. All eight (100%) T1 patients had N3 disease, while of the 53 T2 patients, 30 (56.6%) had N2 and 23 (43.4%) had N3 disease; therefore, of the 61 patients with T1-2 disease, 30 (49.2%) had stage III NPC and 31 (50.8%) had stage IV NPC.

Among the 316 eligible patients, 110 (34.8%) experienced treatment failure and 117 (37.0%) died during the follow-up period. Most first failures occurred at distant sites: 77 (24.4%) patients developed distant metastasis and 37 (11.7%) developed locoregional relapse (of whom four had concomitant distant failure).

### Evaluation of surrogate endpoints for OS

#### Prentice’s Criterion 1

**Figure 1:**
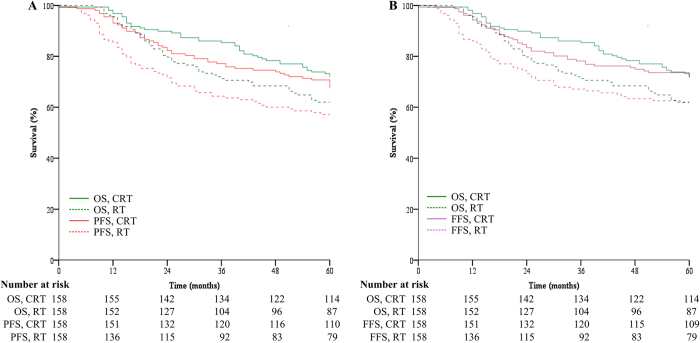
Kaplan–Meier (**A**) overall survival (OS) and progression–free survival (PFS) and (**B**) OS and failure–free survival (FFS) curves. The survival curves were truncated at 5 years. The number of patients at risk in each group is given below the graph. CRT, chemoradiotherapy; RT, radiotherapy.

The true study endpoint, 5-year OS, was 62% in the RT group and 72% in the CRT group (*P*_log-rank_ = 0.038; Fig. 1). The adjusted HR for CRT was 0.66 (95% CI, 0.44–0.97; *P*_Cox_ = 0.036). This translates to a 34% relative reduction in deaths after CRT compared to RT. Therefore, Prentice’s first criterion, that the treatment is prognostic for the true endpoint, was met.

#### Prentice’s Criterion 2

Statistically significant treatment effects were observed in terms of 2-year and 3-year PFS (for 2-year PFS: HR = 0.55, 95% CI 0.35–0.89; for 3-year PFS: HR = 0.57, 95% CI 0.37–0.86), and also 2-year and 3-year FFS (for 2-year FFS: HR = 0.55, 95% CI 0.33–0.89; for 3-year FFS: HR = 0.57, 95% CI 0.38–0.91; Fig. 1, Table 1). However, treatment arm was not a significant prognostic factor for 2- and 3-year D-FFS or LR-FFS, though treatment arm had a marginally significant effect on these outcomes (Table 2). Therefore, Prentice’s second criterion, that treatment is prognostic for the surrogate endpoint, was only met for 2-year and 3-year PFS and FFS.

#### Prentice’s Criterion 3

In both the 2-year and 3-year evaluations, failure or death due to any cause, and failure at any site within ≤2/3 years had a significant impact on 5-year OS (all *P*_Cox_ <0.001; Table 3). As treatment arm was not prognostic for distant and locoregional failure within ≤2 or 3 years, Prentice’s third criterion was not tested for these endpoints. Prentice’s third criterion, that the surrogate endpoint is prognostic for the true endpoint, OS, was met for 2- and 3-year PFS and FFS.

#### Prentice’s Criterion 4

To assess this criterion, the first hypothesis tested whether 5-year OS was independent of treatment in patients who suffer failure or die due to any cause within ≤2/3 years, or suffer failure at any site within ≤2/3 years. The second hypothesis tested whether 5-year OS was independent of treatment if none of the abovementioned events occurred within ≤2/3 years (Table 4). The effect of treatment on 5-year OS was not significant for either hypothesis (all *P*_Cox_ >0.10; Table 4). These findings suggest the full effect of treatment on 5-year OS can be explained by the surrogate endpoints PFS and FFS, independently of treatment; therefore, Prentice’s fourth criterion was satisfied.

### Strength of associations between surrogate endpoints and 5-year OS

The degree of association between the surrogate and true endpoints were measured using the Spearman rank correlation coefficient (ρ). The coefficient was moderate for 2-year PFS and 5-year OS (ρ = 0.73) and also for 2-year FFS and 5-year OS (ρ = 0.69); however, it was 0.84 for 3-year PFS and 5-year OS, and 0.78 for 3-year FFS and 5-year OS. Therefore, compared to 2-year PFS/FFS and 5-year OS, a relatively strong association existed between 3-year PFS/FFS and 5-year OS, especially between 3-year PFS and 5-year OS.

## Discussion

This study is a secondary analysis of a prospective randomized phase III trial, in which the treatment effects of CRT and RT were compared in locoregionally advanced NPC. The purpose of this analysis was to apply Prentice’s surrogacy criteria to evaluate which secondary endpoints could be useful surrogates for 5-year OS. Both PFS and FFS at 2 and 3 years were consistent with all four of Prentice’s criteria, while D-FFS and LR-FFS were not significantly different between arms at 2 and 3 years. Moreover, compared to 2-year PFS and 2/3-year FFS, 3-year PFS had a relatively strong association with 5-year OS (ρ = 0.84). Thus, 2 and 3-year PFS and FFS, especially 3-year PFS, could serve as valid surrogate endpoints for 5-year OS and may enable early assessment of treatment effects in patients with locoregionally advanced NPC.

According to a recent population-based study in China, NPC has a relatively longer natural history than other aggressive malignancies such as lung cancer, gastric cancer, liver cancer and esophageal cancer^11^. Five-year OS for locoregionally advanced NPC ranges from 58 to 75%^12^. Identification of surrogate endpoints could enable prediction of the effects of treatment on long-term survival at earlier time-points; in future trials, this could reduce the follow-up period and accelerate the development of therapeutic regimens. Surrogate endpoints measured at later time-points are less useful as they become closer to the final endpoint. However, a very short period of observation may lead to a loss of events and lead to difficulties in observing significant differences and inaccurate prediction of the true endpoint. In our analysis, both D-FFS and LR-FFS measured at 2 and 3 years did not obey Prentice’s second criterion and are not suitable surrogate endpoints. Though several meta-analyses^6^,^13^,^14^ have demonstrated CRT can effectively reduce distant and locoregional failures, these studies included many patients from a number of trials, which makes it relatively easier to obtain a positive result. In general, it is difficult for a trial to recruit so many patients. Besides, there might not be sufficient events for D-FFS and LR-FFS at early time-points, which explains our failure to validate 2- and 3-year D-FFS and LR-FFS as surrogate endpoints.

Generally more PFS and FFS than D-FFS and LR-FFS events occur, which increases the statistical power for PFS and FFS and enables small randomized trials to be performed. This study demonstrated both 2- and 3-year PFS and FFS were valid surrogate endpoints for 5-year OS. The rank correlation coefficient for both surrogates weakened with reduced follow-up duration, with the highest coefficient observed for 3-year PFS and 5-year OS. As PFS was defined as death or failure at any site, the overlap between the definitions of PFS and OS may account for the superior predictive ability of PFS compared to FFS. Thus, it may be appropriate to use 3-year PFS to predict long-term OS. Overall, the use of 2/3-year PFS and FFS as surrogate endpoints could reduce the number of patients that need to be recruited and shorten the duration of clinical trials by permitting earlier reporting of the results, thereby reducing the cost of developing effective therapeutic strategies for locoregionally advanced NPC. Furthermore, use of the validated surrogate endpoints would reduce the risk of abandoning potentially effective new treatments when analysis of OS is complicated by subsequent treatments after disease progression.

Several limitations of this study should be acknowledged. First, the limitations of applying statistical methods such as Prentice’s criteria to validate surrogate endpoints need to be noted, and caution should be taken when interpreting these results^15^. Prentice’s fourth criterion is very difficult to assess as it is formulated in terms of an equivalence setting; meeting this criterion is not necessarily definite evidence that the criterion holds^16^. Still, 2- and 3-year PFS and FFS met all four criteria, indicating their validity as surrogate endpoints for 5-year OS. Secondly, patients from only a single trial were included in this analysis. Surrogate endpoint analyses conducted using data from a number of trials could help to reinforce the conclusions of this study. Although data from only one trial were available, the findings of this study are strengthened by the fact they are derived from a relatively large cohort treated at a single institution with long-term follow-up. The trial was conducted using a reliable randomization method with standardized data collection, treatment and follow-up protocols, which should avoid potential sources of selection or other incidental bias. Third, though detection of distant metastasis and locoregional recurrence may be subject to variation depending how often imaging studies are obtained; assessment of PFS or FFS is not as definitive as OS. Also, though D-FFS and LR-FFS were not validated as surrogate endpoints, these endpoints remain useful as they reflect which sites of failure (local or distant) a treatment controls.

A surrogate endpoint can only be validated for the treatment assessed; extrapolation to a therapeutic regimen with a different mechanism of action may not be warranted. Here, we investigated CRT in a prospectively-identified cohort of patients with locoregionally advanced NPC. This CRT regimen has been recommended as a standard treatment option for locoregionally advanced NPC^6^, indicating this analysis is contemporary with a broad clinical applicability. However, we still need to directly verify surrogate endpoints for trials assessing targeted or immunological therapies or trials that include selected patient subgroups.

These analyses represent an initial attempt to explore possible surrogate endpoints for OS in locoregionally advanced NPC on the basis of Prentice’s four criteria; 2- and 3-year PFS and FFS were identified as valid surrogate endpoints for early assessment of treatment effects. Identification of other potential biomarkers, such as Epstein-Barr virus DNA load^17^ and microRNAs^18^, as surrogate endpoints may help to better predict long-term survival outcomes at an early stage before treatment failure occurs, and enable appropriate salvage treatments to be administered in a timely manner. However, surrogate endpoints do not eliminate the need for long-term follow-up, as unexpected late side-effects may not be captured by the surrogate endpoints. Clinical trials should continue to be designed with potential surrogate endpoints as part of the trial protocol, along with OS.

## Additional Information

**How to cite this article**: Chen, Y.-P. *et al.* Potential surrogate endpoints for overall survival in locoregionally advanced nasopharyngeal carcinoma: an analysis of a phase III randomized trial. *Sci. Rep.* **5**, 12502; doi: 10.1038/srep12502 (2015).

## Figures and Tables

**Table 1:** Baseline characteristics.

Characteristic	CCRT + AC group (*N* = 158)	RT group (*N* = 158)
Sex (male)	116 (73%)	120 (76%)
Median age (years)	47 (21–68)	46 (23–68)
Performance status		
0–1	144 (91%)	142 (90%)
2	14 ( 9%)	16 (10%)
Pathology		
Undifferentiated	153 (97%)	153 (97%)
Differentiated	5 (3%)	5 (3%)
T classification^a^		
T1	5 (3%)	3 (2%)
T2	27 (17%)	26 (17%)
T3	84 (53%)	86 (54%)
T4	42 (27%)	43 (27%)
N classification^a^		
N0	27 (17%)	29 (18%)
N1	46 (29%)	53 (34%)
N2	49 (31%)	47 (30%)
N3	36 (23%)	29 (18%)
Staging^a^		
III	89 (56%)	91 (58%)
IVA–IVB	69 (44%)	67 (42%)

Data are n (%) or median (range). CCRT, concurrent chemoradiotherapy; AC, adjuvant chemotherapy; RT, radiotherapy.

^a^According to the AJCC 5th edition.

**Table 2:** Survival outcomes for the surrogate endpoints by treatment arm.

Surrogate endpoint	Events			Survival rates for surrogate endpoints^a^			
	CRT group (*n* = 158), No. (%)	RT group (*n* = 158), No. (%)	Total, No. (%)	CRT group, %	RT group, %	HR (95% CI)^b^	*P*-value^c^
Two-year analysis							
PFS	28 (18)	45 (28)	73 (23)	84	73	0.55 (0.35–0.89)	0.014
FFS	26 (16)	42 (27)	68 (22)	85	74	0.55 (0.33–0.89)	0.015
D-FFS	21 (13)	32 (20)	53 (17)	87	81	0.60 (0.35–1.04)	0.069
LR-FFS	5 (3)	12 (8)	17 (5)	98	92	0.40 (0.14–1.13)	0.082
Three-year analysis							
PFS	36 (23)	56 (35)	92 (29)	77	64	0.57 (0.37–0.86)	0.008
FFS	34 (21)	51 (32)	85 (27)	78	67	0.57 (0.38–0.91)	0.016
D-FFS	29 (18)	39 (25)	68 (22)	81	74	0.66 (0.41–1.06)	0.088
LR-FFS	6 (4)	14 (9)	20 (6)	96	90	0.41 (0.16–1.05)	0.064

PFS, progression-free survival; FFS, failure-free survival; D-FFS, distant failure-free survival; LR-FFS, locoregional failure-free survival; CRT, chemoradiotherapy; RT, radiotherapy; HR, hazard ratio; CI, confidence interval.

^a^The survival rates for the surrogate endpoints were estimated using the Kaplan-Meier method.

^b^Hazard ratio was adjusted for the following parameters using the Cox proportional hazards model by backward elimination: age (>50 vs.≤50 years), sex (male vs. female), T classification (T3–4 vs. T1–2), N classification (N1–3 vs. N0), and treatment arm (CRT vs. RT).

^c^*P*-value based on a chi-square test using the Cox proportional hazards model.

**Table 3:** Surrogate endpoints as prognostic factors for 5-year overall survival^a^.

Prognostic variable^b^	HR (95% CI)^c^	*P*-value^d^
Two-year analysis		
Failure or death from any cause ≤2 years	44.3 (25.3–77.7)	<0.001
Failure at any sites ≤2 years	27.4 (16.8–44.9)	<0.001
Three-year analysis		
Failure or death from any cause ≤3 years	25.8 (16.5–40.5)	<0.001
Failure at any sites ≤3 years	20.2 (13.0–31.2)	<0.001

HR, hazard ratio; CI, confidence interval.

^a^As treatment was not prognostic for distant failure or locoregional failure, Prentice’s criteria 3 and 4 were not tested for these endpoints.

^b^The reference groups had no events ≤2/3 years.

^c^Hazard ratio was adjusted for the following parameters using a Cox proportional hazards model by backward elimination: age (>50 vs.≤50 years), sex (male vs. female), T classification (T3–4 vs. T1–2), N classification (N1–3 vs. N0), and surrogate endpoints (with vs. without events ≤2/3 years).

^d^*P*-value based on chi-square test using the Cox proportional hazards model.

**Table 4:** Effect of treatment on 5-year overall survival in patients stratified by the surrogate endpoints^a^.

	Five-year overall survival^b^		
	CRT group, %	RT group, %	*P*-value^c^
Two-year analysis			
Patients with failure or death from any cause ≤2 years			
Yes	4	7	>0.10
No	88	84	>0.10
Patients with failure at any site ≤2 years			
Yes	4	7	>0.10
No	87	82	>0.10
Three-year analysis			
Patients with failure or death from any cause ≤3 years			
Yes	6	7	>0.10
No	93	93	>0.10
Patients with failure at any site ≤3 years			
Yes	6	8	>0.10
No	92	89	>0.10

CRT, chemoradiotherapy; RT, radiotherapy.

^a^As treatment was not prognostic for distant failure or locoregional failure, Prentice’s criteria 3 and 4 were not tested for these endpoints.

^b^The survival rates for surrogate endpoints were estimated using the Kaplan-Meier method.

^c^*P*-value based on the chi-square test using the Cox proportional hazards model. The following parameters were included in the Cox proportional hazards model by backward elimination: age (>50 vs. ≤50 years), sex (male vs. female), T classification (T3–4 vs. T1–2), N classification (N1–3 vs. N0) and treatment arm (CRT vs. RT).
